# Future Supply Chains Enabled by Continuous Processing—Opportunities and Challenges. May 20–21, 2014 Continuous Manufacturing Symposium

**DOI:** 10.1002/jps.24343

**Published:** 2015-01-28

**Authors:** Jagjit Singh Srai, Clive Badman, Markus Krumme, Mauricio Futran, Craig Johnston

**Affiliations:** 1 Department of Engineering, Institute for Manufacturing, University of CambridgeCambridge, United Kingdom; 2 Pre-Competitive Activities, GlaxoSmithKlineStevenage, United Kingdom; 3 Continuous Manufacturing Unit, Novartis Pharma AGBasel, Switzerland; 4 Process Science and Advanced Analytics, Janssen Supply Group, LLCNew York, USA; 5 Continuous Manufacturing and Crystallisation, University of StrathclydeGlasgow, United Kingdom

**Keywords:** biopharmaceuticals characterization, controlled delivery, controlled release, tableting, processing, polymorphism, bioavailability, chemical stability

## Abstract

This paper examines the opportunities and challenges facing the pharmaceutical industry in moving to a primarily “continuous processing”-based supply chain. The current predominantly “large batch” and centralized manufacturing system designed for the “blockbuster” drug has driven a slow-paced, inventory heavy operating model that is increasingly regarded as inflexible and unsustainable. Indeed, new markets and the rapidly evolving technology landscape will drive more product variety, shorter product life-cycles, and smaller drug volumes, which will exacerbate an already unsustainable economic model. Future supply chains will be required to enhance affordability and availability for patients and healthcare providers alike despite the increased product complexity. In this more challenging supply scenario, we examine the potential for a more pull driven, near real-time demand-based supply chain, utilizing continuous processing where appropriate as a key element of a more “flow-through” operating model. In this discussion paper on future supply chain models underpinned by developments in the continuous manufacture of pharmaceuticals, we have set out;

- The significant opportunities to moving to a supply chain flow-through operating model, with substantial opportunities in inventory reduction, lead-time to patient, and radically different product assurance/stability regimes.
- Scenarios for decentralized production models producing a greater variety of products with enhanced volume flexibility.
- Production, supply, and value chain footprints that are radically different from today's monolithic and centralized batch manufacturing operations.
- Clinical trial and drug product development cost savings that support more rapid scale-up and market entry models with early involvement of SC designers within New Product Development.
- The major supply chain and industrial transformational challenges that need to be addressed.

The paper recognizes that although current batch operational performance in pharma is far from optimal and not necessarily an appropriate end-state benchmark for batch technology, the adoption of continuous supply chain operating models underpinned by continuous production processing, as full or hybrid solutions in selected product supply chains, can support industry transformations to deliver right-first-time quality at substantially lower inventory profiles. © 2015 The Authors. *Journal of Pharmaceutical Sciences* published by Wiley Periodicals, Inc. and the American Pharmacists Association J Pharm Sci 104:840–849, 2015

## INTRODUCTION

The supply chain structure of the pharmaceutical industry in terms of in-bound material supply, production footprint in active processing and drug product manufacture, and downstream supply chain operations has not changed for decades. Despite healthy product margins and progressive improvements in production process control and consequent productivity, the pharmaceutical industry when compared with other process industries, operational performance levels are well below process-industry norms on right-first-time quality, inventory, and service levels. Structural changes to pricing models may, in the future, also challenge this strong margin position, as healthcare providers move to a manufacturing-cost-based pricing model rather than the “value”-driven pricing arrangements of today.

In terms of quality and the repeatability of manufacturing processes, most pharmaceutical firms operate at levels of between 3 and 4σ in terms manufacturing right-first-time, costing the global industry some $20 bn annually.

The current predominantly batch and centralized manufacturing model has resulted in product supply chains, which typically are between 1 and 2 years in length, with a huge associated cost of inventory. The manufacturing assets that most “big pharma” companies have for product manufacture are suited to blockbuster supply, relying on large-scale centralized batch manufacturing plants, located predominantly in developed countries. Current trends in the industry suggest that smaller, more niche volume products will become the norm with fewer blockbusters, within a market demand context where globalization will require the ability to supply multiple geographically dispersed locations, collectively representing a more fragmented product portfolio. It is against this background that we seek, in this paper, to look at the impact of continuous manufacturing on the supply chain. In this context, we go beyond the simple “batch” or “continuous” production process technology choice, but consider how we might migrate supply chains from a “batch” campaign mind-set, to a continuous material flow model, utilizing continuous production processing technologies where appropriate.

The paper considers how a change to continuous processing might transform the industry to a more efficient and adaptive manufacturing supply chain that is being increasingly demanded by institutional payers leading to benefits to end-user patients. This is becoming all the more necessary for the industry as new technologies and affordability challenges require multiple supply chain models that can deliver the drug products of the future. The paper is structured as follows; identifying the scale of the opportunity, setting a vision for future pharmaceutical supply chains and the business models that this future context may involve, how these future networks may be designed, and the transformation challenges that need to be overcome to realize the potential of continuous manufacture. In this transformation context, the dynamic capabilities required to transform the industry will be discussed and how both risk and resilience will feature in the design of future supply chain models.

## SCALING THE OPPORTUNITY

In this section, we consider “What might the benefits of more flexible, responsive continuous manufacturing-based end-to-end (E2E) supply and innovation chains bring to the healthcare/pharmaceutical manufacturing sector?”

In our analysis of current E2E supply chains, we observe the following potential opportunities from moving to continuous process manufacturing:

- Greater product and volume flexibility enabling multiple supply chain models, more tailored to specific market needs
- Significant inventory reduction opportunities through a more responsive E2E SC (Supply Chain)
- Improved quality
- Rapid scale-up post clinical trials, perhaps redefining the nature of clinical trials and subsequent commercial production
- Reduction in the management burden and overhead structural costs they generate in the in the current supply chain paradigm where multiple human to human hand-offs are required to deliver product to patients

These five themes are further developed below, outlining where the opportunity sits.

### Greater Product and Volume Flexibility Enabling Multiple Supply Chain Models, More Tailored to Specific Market Needs

It is now generally accepted that the pharmaceutical industry is progressively moving away from the large volume “blockbuster” drug production model and requires future production and supply chain models that can deliver significantly greater product variety and volume flexibility. Indeed, this may involve product delivery models developed or tailored to serve relatively niche markets where patient populations are significantly smaller than today's norms. This, together with advances in stratified and personalized medicines, will require levels of product customization that make the batch centric production models of today incapable of economically supplying these product varieties (SKUs) at the smaller volumes required, and at the speed increasingly demanded by end-users (patients and payers) without the costly “buffer” of huge inventory.

The potential opportunities for “continuous processing” centric manufacturing supply chains include:

- New capabilities for firms to meet as yet unmet patient needs, by developing capabilities to supply niche markets that are currently uneconomical to serve because of the small product volumes linked to specific patient populations.
- The volume flexibility afforded by continuous processing, unconstrained by batch size, has major implications for materials requirements and inventory, shortening dramatically the shelf-life of products that are often determined by minimum batch volumes.
- Better adaption to market supply and country versions: variety of small markets (in terms of units produced, not value) can drive a substantial accumulated volume. Impact of individualized medicine can require a variety of strengths that need to be produced, each as multiple country versions. In the batch world of today, this is difficult to handle and negatively impacts on the Cost of Goods Sold, inventory and shelf life requirements, as well as operational costs linked to increased complexity.
- Late customization and deferment models is another alternative supply scenario, where final product definition is achieved in-market within a distributed and geographically dispersed “final stage” manufacturing activity; in this scenario, both upstream and downstream stages may preferentially support continuous process technologies that support “material flow” supply dynamics as opposed to the current “batch” campaign model.

Within medium-to-large volume-scale manufacture, two key questions emerge; the volume-scale where the transition from batch to continuous becomes attractive, and whether the desired volume flexibility may be achieved by various combinations of batch and continuous processing. As continuous manufacturing overcomes the discretization of batch sizes, it opens up a range of volume options not otherwise possible. Furthermore, the development of post-dosing manufacturing capabilities might afford further levels of late-customization when applied to a common base product. If the latter is made continuously, the volume options for “minimum order quantities,” a key criterion in supply chain design, become potentially unconstrained.

One potential application area is in supporting patient “dose flexibility.” Whereas dose flexibility is already a reality in injectable products, oral liquids, and semisolids, where the patient is provided this flexibility, this may also be deliverable in discrete dose formats. For example, liquid forms produced in continuous mode, can potentially provide varying formulations, process controlled to conform to specification. Alternative technologies would be required for solid-dose forms, such as additive production process models, ink-jet styled dose control strategies, or novel “multi-dose” pack-formats and pack-devices to deliver this dose flexibility functionality.

Late product customization, integrated with individualized pack labeling, will be fundamental in supporting potential developments in individualized and personalized medicines. The availability of faster and more flexible supply chains, as enabled by continuous processing, may also enable products and dosage forms that inherently have shorter shelf lives.

### Significant Inventory Reduction with a More Responsive E2E SC

The opportunities for a significant reduction in inventory through continuous processing results from chemical processing models offering reduced process steps and process equipment that provides significant volume flexibility. This potentially leads to substantial reductions in inventory which in-turn enable:

- Moving to more of a “demand-driven” replenishment model rather than the current long-term forecasting approaches with wider opportunities for manufacturing and supply chain integration.
- The ability to operate on substantially shorter lead times for product replenishment by significantly reducing intermediate and finished goods stock levels.
- Consideration of chemical routes involving short-lived unstable intermediates within continuous manufacturing, normally avoided in batch-chemistry, opening up alternative synthesis routes.

For these benefits to be effectively realized, the industry will need to confirm the expected improvement in supply chain robustness and resilience ensuring complete confidence in the supply chain delivering medicines to patients.

### Improved Quality

Continuous processing can lead to substantially less rework, assuming rapid start-up to steady state and that recycling is routinely possible within agreed operating and regulatory frameworks. As consistency is one of the hallmarks of continuous processing, a well-designed and effectively run continuous process can deliver a highly consistent product, leading to lower variance and more reliable performance.

Continuous processing has, among other aspects, one fundamental differentiator from batch processes, which can have a significant impact on the supply scenario. This aspect is process control and the capability of enforcing process conditions at a micro-level, which has a fundamental impact on development processes, on quality and on supply.

Batch processes, for example, operate under the paradigm that the totality of material is transformed in a reactor of some sort, which holds the totality of material all at once. This makes the reactor size dependent on the desired batch size. Reactor size, however, drives the enforceability of process conditions of the entirety of material on a micro-level. An illustration of this is in an exothermic chemical reaction where heat management is critical to controlling the reaction. The heat generation is endogenous to the material and the heat control can only be obtained by cooling the walls of the reactor. Temperature is dependent on the distance of the point of interest to the wall. The larger that distance, the smaller the impact of the wall temperature, in our case, the cooling effect on the reactive conditions, such that local overheating is a real possibility, as even with the best temperature control in the reactor wall, the freedom of the reaction to exhibit local overheating (that is not even noticed) is high.

Similar examples exist for other processes, which may also have complex numerical scenarios involved, aka nonlinearities in material laws and heat flow or property propagation in general terms. Examples include batch crystallizers, wet granulation in shear mixers (high or low), including the fluid bed drying of tableting processes, as the special distribution of properties within a single tablet is not always uniform. In summary, the larger the reactor is (or shall we better say the process equipment to describe in an abstract way everything from a chemical reactor to a powder handling system), the less control we have over the real conditions that transform the material. Consequences of that statement are among others that the quality of the transformation is only loosely controlled, and a robust or in other words forgiving product or formulation is needed to limit the impact of this. Again in our exothermic reaction example with poor control at the micro-level of reactive conditions like temperature and mixing, local overheating may occur and even degrade the product through a deteriorating follower reaction or decomposition. In the batch world, these by-products will be diluted into the entire batch and will raise impurities. Similar effects can be named for almost any other unit operation: in wet granulation local over granulation would come to mind, in tablet formation capping as a consequence of inhomogeneity of process conditions and so on, the list is long. So, in other words, a different scale of unit operation has a fairly high chance of quality attributes being scale dependent. One can even drill this down to a tablet size being the determining factor for the last “material transformation” in the pharmaceutical delivery chain, the dissolution in the patient's stomach. The consistency potential of continuous processing is thus significant both in terms of quality but also as set out below the opportunities for more rapid scale-up.

### Rapid Scale-Up Post Clinical Trials

In terms of the challenges in commercial scale-up of continuous processes, once the manufacturing process has been established within the clinical trial regime, these are widely recognized as being significantly smaller and less expensive than for traditional batch processes.

The cost of bringing products to registration can be significantly reduced when using continuous manufacturing for the design of experiments (DoE) during development to support a quality-by-design (QbD) filing. By way of exemplification, GSK have demonstrated significant reduction in scale-up time and cost during development by switching from batch to continuous granulation.^1^ When a product transfers to commercial manufacture, it is anticipated that this will achieve reductions in operating costs, work in progress, and footprint. This switch from batch to continuous granulation also provided evidence of significantly reduced variance in the size distribution of the granules. There are also potentially reduced material requirements for scale up in continuous processing trails. In a batch-based operation, the scale is defined by the size of the process equipment that is used. It defines the amount of material that is exposed to a homogeneous application of processing conditions. Any variation of processing conditions requires the production of this quantity of material at the given set-point. This multiplies the material consumption per set-point with the batch size and hence leads to huge material consumption to prove the validity of different set-points of the processes. In continuous processing, the variation of set-points can take place “on the fly” and hence allows a much faster set-point screening. It practically replaces the minimum amount of material per processing condition from the batch defined amount to an amount that is given by the transient time it takes to change from one set-point to another. These can be significantly smaller in a continuous processing setting and hence the amount of materials required for an array of set-points can be substantially reduced. This can be seen as an easier and less costly scale-up model.

A specific example of reduced material requirements for scale-up is in the quantity of API(Active Pharmaceutical Ingredient) required to fully develop and scale up to commercial scale. In a batch process, quantities are typically not available at the phase II stage of development. Performing multivariable factorial DoE (a key component of QbD development) using large-scale batch processes is time consuming because each data point in a multi-step process that could take several days or weeks to generate. In contrast, a comprehensive DoE with multiple data points could be performed in less than a day with continuous manufacturing.

### Reduction in Management Overhead Costs

As discussed earlier, significant managerial costs, often “hidden” in company manufacturing or supply chain overheads, are driven by the managerial resources required to operate the supply of products through the manufacturing supply chain. A more continuous flow operating model would require systemized linkages across production and supply operations, minimizing human to human hand-offs, and driving the need for better demand signal detection through the E2E supply chain. Currently, these overheads “allocated” to products may equate to around 50% of the product cost, and although constitute a multitude of cost factors, are primarily linked to the combination of expensive and under-utilized assets and associated depreciation charges, but also the management costs involved in the oversight of these complex interactions.

In quantifying the opportunity, Table 1 provides an assessment of the potential scale of the opportunities *across the theme areas* that can be targeted over the medium term.

**Table 1:** Scaling the Potential Opportunities Across the End-to End Supply Chain

End-to-End Supply Chain Opportunity
Reducing inventory within primes from >200 days to <70 days
Manufacturing—cost of quality, achieve >5*σ*, right-first-time
1–2 years inventory days of supply—opportunity to reduce up to 50%
Reduce cycle time by half (starting materials to packed product)
Reduce drug development cost, currently at $1.15 bn/drug,^2^ by 10% (cost to market)
Enhance flexibility and service to patients, improving both patient service and compliance through more demand driven responsive supply chains
Reduction in management overheads, reducing the manual interactions in the oversight of batch-campaign operating models, through enhanced flow-through supply concepts

The potential benefits longer term can be even greater than those set out above but recognize the transformation journey is unlikely to be realized quickly because of the factors set out in later sections in this paper. Indeed, current industry performance does not represent an optimized batch model—far from it, and some observers will question whether an industry that is unable to run what many would regard as simpler batch processes effectively, can deliver efficient continuous manufacturing processes. However, a key difference is that continuous processes impose disciplines that are optional in a batch model.

## CONTEXT/FUTURE VISION

Here, we consider how might the healthcare/pharmaceutical industrial ecosystem evolve in a predominant continuous manufacturing innovation and supply model in terms of changes to industry structure, adoption of enabling technologies, and the provision of new products and services to smaller patient group populations? Potential future developments within the medium term timeframe include:

- Patient (rather than health provider) centric supply chains that support multiple value and supply chain configurations, coexisting and providing different, often more localized and dynamic replenishment models.
- Simplified supply chain operations with less managerial oversight and regulatory sanction.
- More responsive production and distribution models that can support rapid replenishment driven by the emergence of patient management diagnostics and “Apps,” medical devices, and supply chain integrating IT systems.
- Reduced capex and operating costs, and volume flexibility, afforded by continuous processing supports more geographically distributed production and supply networks, closer to patient demand.
- Process-control-based quality and regulatory assurance becoming established mechanisms, supported by advances in Process Analytical Technologies (PAT) that provide real-time data on product and process consistency during production, and used to quality assure product direct into the supply chain.
- Easier supply to smaller patient groups (by strength, by country) including earlier access to commercial scale materials for patients, as scale-up requirements become significantly less onerous. The reduced development timelines can increase the profitable supply time for innovators, allowing more development resources for the overall enterprise (assuming a supportive regulatory environment).
- More localization, enabling more dynamic closed loop control, with control parameters set upstream based on downstream measurements.

In terms of future scenarios, we might imagine the progressive emergence of cheap robotics and microprocessor control as well as advanced, but cheap, sensor systems supporting new models whereby complex molecules and bio-pharms are synthesized on small modular platforms, and numbered up to scale (as required). The decentralization of manufacturing that would occur would be unprecedented in the chemicals industry.

Another example of future implementation paths is the use of hybrid 3D printing systems to produce configurable flow reactors with sensing, actuation, reaction processing, purification, and so on. 3D printing is a process where objects can be fabricated layer by layer, or part by part, allowing computer design and easy customization of architectures. 3D printers come in several flavors, as well as high-end commercial systems and affordable, open source, user customizable devices. For instance, Cronin et al. have shown it is possible, using open-source 3D printers, to “hack” plastic laboratory ware. However, the development of this “hackware” is allowing the development of hybrid devices where the 3D printer is used not only to construct a test tube for a chemical reaction, but also deploy/pump the chemicals into the test tube for the reaction and also customize the test tube to allow certain reactions to happen in different ways. This could even be extended to biologics by printing bio-reactors. The 3D printer acts in two ways. Firstly, it can be used to fabricate the plastic-ware, or “reactionware” (the “flow system” in which the chemistry is carried out), and secondly we can use the printer as a robot to move chemicals around to do chemical reactions. The 3D model can particularly support the deferment and late customization supply models within secondary processing, by allowing near-to-end market final processing and customization.

The potential realization of the “modular” chemical factory would require a new set of standards allowing modular interchange from a physical, chemical, electronic, and software point of view. The natural consequence of this could enable the retasking of the “factory” to produce new chemicals or drugs on-demand with zero extra capital cost or investment. This vision requires a radical new integration of chemical systems and synthetic methodologies developed within this new paradigm. The ultimate outcome would be the development of largely software-only manufacturing work-flows whereby the physical system could be reconfigured electronically. (This is similar to peptide or DNA synthesis today but DNA synthesis is an order of magnitude more reliable than peptide synthesis due to the combinatorial problem of conditions for coupling and deprotection as well as purification.)

Again the move to more niche products, possibly with local supply will be enabled by smaller, flexible manufacturing operations. The cost of build when comparing with batch for scale manufacturing plants is potentially reduced by >30%, with physical footprints reduced by >25%, cost of operations reduced by >30%; these modest quantifications of potential benefits are based on successful continuous manufacturing examples already implemented.

At the personalized medicines level, personal “pill” fabrication may have a rather novel application for the consumer. We might imagine a patient that has complex medical issues requiring multiple drugs with multiple dose variations over time. Remembering to take the correct drugs at the correct time is an increasing problem. The local use of “pill-printers” that would simply combine pre-formulated version of the drugs together in either a liquid or solid form using a liquid or powder handling robot into a single dose. The “printer” would be programmed with the prescription of the patient and the drugs mixed together in a binder matrix and then formed into the pill. This could have obvious benefits for the patient in terms of adherence to treatment regimes, improving compliance especially for elderly patients with complex medical conditions.

Final delivery models to the patient could by-pass the current specialist distribution and pharmacy network with direct delivery models, already developed in prototype packaging equipment packing halls, able to serve patients directly with individually named product prescriptions, with multiple products filled on the same line with accuracy levels exceeding the manual checking undertaken within current “pharmacy” models.

Experience from other industries suggest this type of transformation to more flexible localized operations based on changing process technologies and more customized solutions is possible. Transformation examples with analogous comparisons with sector level transformations in other industries include: computer-assisted processing and control in aircraft, decentralization of the printing industry, and transport.

### Computer-Assisted Processing and Control in Aircraft

The E2E continuous processing supply chain will be per se technically very complex, most likely more complex than the current batch model. However, clever use of computerized procedures will enable a better management of these procedures. An analogy with aircraft is that the Airbus A380 is more complex to fly compared with a 1950s prop plane. However, modern control systems can stabilize flight dynamics, navigate, and control the system much better, such that crew can reliably fly this plane and operate it more economically such that mass tourism is now possible, whereas in the 1950s, it was a very much a luxury. The inherent complexity in continuous processing will drive adoption of computer-assisted processing, and greater scrutiny in quality management. We can expect significant benefits to arise from this intense computerization. In chemical synthesis, the emphasis may be on liquid processes instead of solid/liquid dispersions that are easier to control and handle. The processes need to become simpler and more robust in hardware and the higher demand for control can be accommodated in software. This gives rise to a common, and highly compatible, hardware installation and greater impact on software/control. Once accomplished, technical transfers can be as simple as sending data.

### Decentralization of the Printing Industry

The change in supply network structure in moving to continuous processing can be seen as a bifurcation point, as although technically more complex at a processing level compared with the batch paradigm, there is substantial opportunity for significant automation. A historic analogy illustrates this point. Printing in Gutenberg's time was a relatively simple process at a technical level. Even in the 1970s, it remained a fairly simple process albeit complicated by the addition of the mechanical features of mass-production. The printing process as such was still a simple shaping exercise of a stamp of some sort, inking it somehow, and transferring that ink to a sheet of paper. Today's laser printing is technically much more complex, but it is universally available, even when consumers do not have the slightest idea as to what is going on inside the printer itself.

The overall global consumption of paper has risen significantly since decentralized printing became available, despite the increased complexity of the technology. It is useful to note that the first laser printers in the 1970s were research institution type activities and yet are now a ubiquitous commodity.

In this example, complexity increases substantially in the initial phase requiring technical specialization with production capacity in centers of expertise. However, as the technology matures, production is progressively decentralized and requires smaller footprints at each production site. Decentralization will then drive supply chain topologies in different ways, as QA aspects are managed through in-field real-time data, or in the case where intervention is needed, the “cloud”-based operator electronically sends the necessary (software) upgrade. Today, the printing industry is highly decentralized, with printers now regarded as consumer goods. The flexibility of the print-on-demand decentralized model has outperformed the per se much faster high-speed printing technology of the traditional print-shop. A similar evolution, of decentralized production with software-based QA and upgrade interventions may also evolve within the continuous manufacturing model.

### Transport

In commercial road transport, vehicles are able to take a variety of routes and select multiple stopping points. Vehicles themselves can have many operating states; traveling at speed, stationary with engine running, stationary with engine turned off, and so on, providing flexibility on delivery route to be taken and the ability to adjust operating costs throughout the journey. In an airplane however, one must operate at a minimum speed, and after take-off typically fly at a predetermined speed and route. Problems during the flight's trajectory must be corrected during motion with limited opportunities to “take-stock” and make unplanned stops. Both road and air transport modes offer opportunities and limitations, and selecting the correct mode of transport, or combinations of the two modes is determined by the context and flexibility required. There is no doubt that different transport requirements, in terms of distance to be traveled and number of drop-points will favor air or road transport in different ways, and a smart combination of both approaches will best support a diverse supply landscape.

These future supply scenarios highlight how batch and continuous operating models have evolved in other industries. These examples serve to demonstrate how decentralized and highly controlled process technologies have influenced the evolution of supply chain models in other industries and the mind-set required to make such changes a reality.

## CAPTURING VALUE ACROSS THE E2E SUPPLY CHAIN

In this section, we explore the potential new business models and value propositions that might emerge from a more integrated E2E continuous manufacturing-based supply chain and whether the existing infrastructure meets the needs of the changing product portfolio. Example developments to capture value across the supply chain include:

- Emergence of products supported by Medical Diagnostic Devices enable the capturing of product demand requirements directly between patient and drug provider.^3^ In a highly networked scenario, the supply chains would operate as reconfigurable and adaptive networks that are IT enabled responding to demand fluctuations, linked to remote Patient Diagnostic and Management Systems.
- Technology Convergence; between and within medical technologies that support new (more integrated and patient centric) product and product-service solutions that are more effectively delivered through multiple supply chain models including continuous processing-based supply.
- The potential development of personalized packs described earlier, through technologies that support late customization of products, and novel packaging solutions that facilitate patient compliance and adherence, such as multi-product personalized pack-solutions.
- Decentralized supply models; current batch practice to develop sophisticated scale-up scenarios often involves developing forgiving materials or reactions and as a last resort to widen the quality specs, and locking them as late in the development process as possible. Continuous processes however slice the process conditions along a time axis and hence allow much smaller distances between process conditions as enforced at the boundary of our controllable space and the entirety of the material. This leads to the need to not only know better about the process, but ability to control at a micro level with consequences for improved quality. The other consequence is that the equipment as such is never holding the entirety of material all at once and typically is not only from a reactor room perspective but also from a footprint perspective significantly smaller. Consequently, the process equipment is smaller, the development process is technically more complex but gives better understanding sooner in the process and hence opens the path to much more decentralized supplies for commercial supply scenarios (but also for late phase development scenarios where the supply aspects becomes inherently more important over the “create” aspects of R&D). Whereas for a classical batch regime, this typically involves a monolithic supply center, the continuous paradigm opens the opportunity of developing the fundamental process understanding earlier in R&D. The quantities of materials needed to develop a higher level of process understanding is reduced and then the scaling becomes much less of an issue, if there is a scaling needed at all. Procedurally speaking, the technical transfer into a continuous supply center can take place sooner in the technical development timeline, or if the responsibility is transferred by the regulatory status of the project (“R&D hands over to TechOps at Phase III supply manufacture”) in other words, with fewer development efforts.
- Risk Transfer and Commercial scale-up; if the product volumes are significantly smaller as compared with a blockbuster scenario, then the phase III supply and the launch supply have a chance to be on the same process equipment and even a sustainable commercial supply can be organized in a significantly smaller decentralized supply scenario avoiding risky technical transfers. Risk reduction is plausible as the amount of process enforceability at a micro-level is significantly better giving fewer degrees of freedom for things to go wrong upon site transfers. It needs to be understood though that the technical (engineering) complexities of a continuous production are significantly higher in the design and operational phase.
- Reconfigurability of assets: although continuous process engineering and science will drive more complex processes, they will provide opportunities for better process control, better quality, and smaller footprints, leading to smaller supply centers and eventually faster transfers into them. Taken one step further, the localization of this value generation allows a much greater flexibility in terms of physical assets as a smaller plant is easier to relocate, and the driving factor becomes much more the availability of human brain-power at these dispersed locations to manage the inherent process complexities.

Existing infrastructure is unlikely to support the needs of the evolving portfolio and emerging supply models; fewer blockbusters, more niche products, stratified/personalized medicines. Nor is that infrastructure likely to be in the right place with changes to markets, products, and scale. With the emerging markets playing a bigger role in the future thought needs to be given to how they are effectively supported and how this might impact changing industry structure from both a geographical distribution perspective, and asset ownership with contract manufacturing models providing specialist capability and capacity. The potential of reduced inventories and work-in-progress represents perhaps the greatest opportunity for value creation with potential to take out up to 1 year of inventory across the extended supply chain.

## DESIGNING THE E2E SUPPLY CHAIN

Here, we consider the desirable future product, process, and supply network configuration models in a highly continuous manufacturing innovation and supply model. It is perhaps important to note that we envisage multiple supply chain models with configuration scenarios that include:

- Geographically dispersed production networks, supported by more repeatable continuous-based production processes, and offering significant volume flexibility with a flow-through demand driven supply dynamic, progressively replacing the “batch campaign.” Unit operations will involve fewer production steps that change production dynamics from multi-stage multi-location to single location processing.
- Multiple supply chain models that support different levels of geographical reach, with, for example, centralized supply solutions feeding late customized/consolidation models; or alternatively dispersed supply models that support local near-market replenishment models.
- Fragmentation of the downstream supply chain with new “actors” emerging providing specialist services and operating within agreed operating models.
- Localization in small markets is enabled by small continuous lines; the “Factory in a box” is one scenario but so are smaller more standard factory operations that are substantially less capital, labor, and energy intensive providing more resource-efficient sustainable operations.
- Manufacturing “on-demand” with less inventory enabled by continuous lines, which are very well controlled at steady state—reducing the uncertainty of current manufacturing and forecasting processes.
- Continuous processes that enable closer coupling of API and Drug Product operations. However, the need for buffers of intermediate materials should not be wholly discounted. From a quality perspective, control of particles at API should enable more reproducible drug substance manufacture.
- Looking to the future, 3D printing-based supply models enabling local manufacture for a patient-specific drug as part of future developments in personalized medicine.

The transformations will require changes to the roles of existing industry players supporting these more patient-centric demand driven supply chains. From an equipment perspective, improved sensor and control systems to match up to a plug and play approach on a particular manufacturing site, potentially rolled out to across multiple locations. In this scenario, a number of small flexible factories controlled centrally in their operation may support Intellectual Property control and quality assurance while having geographically dispersed physical manufacture.

## RESOLUTION ACTIVITIES (AND CHALLENGES)

A number of global initiatives have been commissioned to take on elements of the transformation challenge. For example, in technology development, programmes are already underway that are required in continuous manufacturing. These include a UK Centre for Continuous Manufacturing and Crystallisation (CMAC), a US-based Novartis-MIT Center for Continuous Manufacturing, the US Centre for Structured Organic Particulate Systems (cSOPS), Ireland SSPC (Synthesis and Solid State Pharmaceutical Centre), various European consortia, and several prototype equipment developments (e.g., UK CMAC Research Partnership Investment Fund). More recently, a new initiative to developing an E2E Supply Chain “eco-system” that considers technology developments (including continuous processing developments) within new supply chain models and the appropriate regulatory regimes required at an industry sector level have been commissioned (UK ReMediES project). These industry-research (and regulator engaged)-based consortia initiatives are enabling sector-wide “pre-competitive” collaborations to support and accelerate transformations to continuous processing.

A key challenge for these teams is in identifying specific product groups where continuous process manufacturing is attractive. Initial research suggests that we should not assume immediately that a SC based on continuous is more flexible and responsive, and that moving an existing product into a continuous (drug product) system may require significant development work. On the development side, we should require much less API for DoEs, engineering runs and validation runs, which will support experimentation where material cost or scarcity is an issue. Another key feature of Continuous Manufacture is that it converts the transformation processes from a strictly stepwise and multiple unit operations-based approach into a world of constant flow of energies and materials. This eliminates the necessity to have holding points before and after each unit operation and eliminates the requirement for the materials to have the ability to survive the holding point without compromising quality. An example is where unit operations are broken up into different geographical locations, to take advantage of duty and tax regimes requiring the shipping of intermediates; these business models constrain the freedom of the process designer and make certain routes impossible, which might otherwise be feasible in continuous manufacturing. On the other hand, in a continuous sequence of processes that are always dynamic, there are limits to possibilities for rework. Also, there is no easy route to stopping a process mid-stream, make a decision, as it is always in flow. Mastered adequately a continuous process offers unique benefits but is not the solution for all cases.

## TRANSFORMATION CHALLENGES

Finally, we explore the major transformational challenges (behavioral, technological, regulatory, etc.) that continuous-based manufacturing and supply models need to overcome. Four key areas are identified regarding transformation to continuous processing, namely:

- Fostering a multi-disciplinary approach across technical and manufacturing disciplines, including requirement for better connectivity between discovery, development, and manufacturing organizations.
- Technology integration across Pharma and Bio-Pharma supply chains including diagnostics to enable patient centric supply chains.
- Derisking investment decisions and overcoming barriers.
- The role of policy across pharma supply chains.

These are explored with recommendations for industry, supply chain practitioners, academia, and regulators and where appropriate, comments from the literature included.

### Fostering a Multi-Disciplinary Approach across Technical and Manufacturing Disciplines

#### Changing the Mind-Set

Changing the mind-set of industrial and institutional professionals, such as regulators, manufacturers, and process engineers, to continuous processing and a retraining of staff will be essential for achieving substantial transformation. At a technical level, this will require developing better capabilities both in continuous synthesis and in the design of continuous drug product manufacture, right through to downstream pack and distribute technologies that can accommodate product variety and flexibility.

#### Transforming Control Regimes

Sampling and testing aliquots of material to confirm quality and manual feedback loops in various Quality organization setups will not be possible nor adequate in continuous manufacturing. If the continuous processing needs the process dynamics to be controlled automatically, as in the flight dynamics of modern flight control systems, the oversight in the field will not be the paper screening of Food and Drug Administration's field inspectors but the approval of the control system and the assurance that the operational parameters are as intended. The quality evidence provided in today's paradigm is either based on manual or simple computerized systems because the integration across systems is according to discipline not according to products. For example: an analytical LIMS system manages all chromatographic data within an organization. The complexity per data entry (meaning per sample) is simple, but because the integration is vertical along all such procedures within an organization, the sheer amount of similar data is overwhelming and here the complexity starts (operator, equipment tag, reagents, injections, sample number, etc.). In the laser printer case, an integrated Photodiode can measure the deposition density of the toner in-line, its data is only used to control this particular printer at the given optimal time-point and guarantees the optimal quality of the printout. Occasional verification is sufficient to verify proper function of all systems and the self-surveillance of the system can help to manage operation.

#### Tackling Organizational Inertia

The current modalities within many large firms in established industries, particularly in those that are highly regulated and technology intense, has involved “committee-based” decision making, often through multi-layer matrix organizations. It has been suggested that this has driven a risk avoidance and tick-boxing culture at a functional level, which promotes incremental innovation despite long product life cycles, at the expense of genuine cross-functional radical innovation. Regulatory contexts inadvertently lock-in these behaviors and preference to established processes. However, in some organizations, we are now witnessing the creation of “autonomous multi-functional teams,” with substantially more devolved responsibilities, to drive more radical transformations—teams that are given the resources, timeframes, and mandates to deliver. Although these are relatively new developments, examples from both the aerospace and pharmaceutical sectors, who both exhibit similar organizational characteristics, partly driven by their industry structures, product architectures, and regulatory frameworks, suggest the “continuous processing” models will require such multi-functional teams to develop specific supply chain models. These multi-functional teams have the opportunity to enhance connectivity between discovery, development, and manufacturing organizations, particularly important in large pharma. In addressing these organizational silos, which are often functional and discipline based, we can encourage the breakdown of unhealthy sub-cultures (that can promote incrementalism and silo behaviors), to take on more challenging cross-functional targets. This will involve developing a new crop of technically based leaders, working within both their own organizations and external parties. Reconstructing industrial “systems” will involve new partnering models with external players focused on delivery against system outcomes. Industry evolution more broadly may result in refocusing industrial activity of the main players on specific elements of the value chain; restructuring activities on value adding activities, perhaps involving mergers and acquisition of firms that operate across the supply chain where vertical integration is critical to product/service delivery.

Interestingly, as current processes and trends move to demands for an ever growing granularity of control per individual control event, the increasing complexity of products and technologies will challenge the existing approach of batch-lot control with its technical limitations, driving firms and regulatory agencies to a system change for the next generation of products. This change in paradigm emerges surprisingly perhaps from the combination for more assurance within the context of increasing complexity, requiring more “systems” cross-functional approaches to quality assurance and new product introduction.

The organizational issues raised here are further explored in the white paper by Krumme et al.

### Technology Integration across Pharma/Bio-Pharma Supply Chains Including Diagnostics to Enable Patient Centric Supply Chains

Integrated product and product-service replenishment models, driven for example by remote diagnostics or near real-time demand signals, will require technology advances that can enable/drive more patient (or institutional user) centric supply/demand models, reducing the reliance on intermediaries. The product categories, patient populations or therapy areas where tightly coupled supply chains might emerge will inform the technology requirements across the product-process-supply domains. Key criteria will be product volume and variety, volume uncertainty and lead-time requirements.

The emergence of new supply models will require policy and regulatory advances that support more direct supplies.

Within the process technology choices during various stages of industry transition, the hybrid batch/continuous models that might progressively support change may be a key consideration in the “road map” to continuous manufacturing and supply. These industrial transition points will themselves have critical dependencies on a number of new technologies, such as better analytical systems, new catalysts, new enzymes, novel control systems, and so on, which in their various combinations will be required to drive success.

At the molecular level, these developments will not only require a rethinking of current molecular discovery to scale up processes, but also require engineers, chemists, and software designers to work together in new ways. Conceptually, organic chemists often work out how to make their target molecule by working backward and reducing the complex molecule to simpler ones step by step on paper, only to reverse the process in the laboratory and build the molecule up. An example of such multi-disciplinary activity is the potential of molecular discovery, scale-up, and crystallization—these processes are being pioneered in the Cronin lab to try and develop this chemistry into “reactionware.” This is not so different from the development of continuous processes from batch to flow. The difference with “reactionware” is that the scaling of the system is much easier and faster because of the ability to rapidly prototype the flow systems using plug and play plastic modules. Of course, we propose going several steps further and using standard modules that can do advanced operations such as separations, crystallizations, and forming composite or formulated products. If the units are cheap, scale up via reactor numbering up is potentially transformative in terms of cost, time, and configurability and mobility.

### Derisking Investment Decisions and Overcoming Barriers

Managing the uncertainty and risk of novel processing routes in Clinical and Commercial supply chains will be critical within any industry adoption of continuous technologies. This calls for industry wide pre-competitive activities to derisk projects and build industry capabilities together with institutional players and regulators.

Another key requirement is lowering barriers to entry—through shared facilities and infrastructure, PAT capability advances at sector level that provide product-process quality assurance, and the proactive development of appropriate regulatory contexts.

Any new technology carries opportunities and risks. In the originator's pharmaceutical business, the main risk is the approval of the compound, driven by the success of the clinical program and the convincing power of the dossier. For a single compound, this is a complex function of a variety of factors, some of which are better manageable than others. The performance and properties of the molecule on the receptor is one element, the biopharmaceutical adequacy of the drug product to the kinetic properties at the receptor and the route to get there is the other and at the end of the day, the feasibility and robustness of distinct process trains for a particular drug product design is of the essence. The magnitude of the risks is typically much larger on the clinical side for originators and hence the focus on secondary risks needs to be minimized. This can be accomplished by a variety of approaches using:

- Technology platforms that are applicable to multiple projects
- New technologies only late in the clinical programs or as life-cycle management tools
- New technologies in a dedicated spin-off that offers the technology for the industry as a whole and hence spreads the risks across multiple products and companies

To value the opportunity and risks adequately, one should not solely consider the technology platform and the success of a specific product or clinical performance, in fact they mostly have nothing to do with each other, other than the fact that a platform has been picked for a particular program. Instead, it is essential to understand what a particular platform delivers in terms of functionality, cost, timelines, and robustness, and quantify those factors. For continuous processing, this reduces the risk to the pure technical risk and other aspects that drive in the long run the success of the process technology on its real merits.

### The Role of Policy and Regulatory Regimes across Pharma Supply Chains

Societal expectations, in developed and emerging markets, will increasingly demand more affordable and/or specialized products available to those who need them.

Institutional pressures on affordability and the demographic impact on national health budgets are expected to drive more efficient supply chains and business models that no longer tolerate the inventory buffers of today. However, the transition to more efficient supply models will require institutional partnerships (government, regulators, and research bodies) with technology and industrial players.

From a regulatory perspective, we anticipate process engineering, analytical methods such as spectroscopy, and data analysis and statistics become progressively more important. Real time access to data and data analysis become the norm, with large-scale sampling and dynamic control methods influencing the regulatory paradigm.

## CONCLUSIONS

In this discussion and review piece of industrial and academic perspectives on the future supply chain models that might be underpinned by developments in the continuous manufacture of pharmaceuticals, we have set out:

- The significant opportunities to moving to a continuous manufacturing supply chain operating model, with substantial opportunities in inventory reduction, lead-time to patient, within radically different product assurance/stability regimes.
- Scenarios for significant decentralized production and supply models producing a greater variety of differentiated products with greater volume flexibility and opportunities for rapid scale-up post clinical trials.
- Production, supply, and value chain footprints that are radically different from today's monolithic and centralized batch manufacturing operations.
- Clinical trial and drug product development cost savings that support more rapid scale-up and market entry models, with early involvement of SC designers within New Product Development.
- The major supply chain and industrial transformational challenges that need to be addressed.

Although the potential benefits against current batch performance benchmarks are significant, identifying the product supply chains where benefits might be most attainable is complex and the transformation journey far from straightforward,^4^ with future archetypes including hybrid batch-continuous scenarios.^5^

Benchmark studies should also consider improvements to current batch operations which operate far from optimal levels. Indeed, some industry observers will question whether the pharma sector can effectively implement these more technically complex continuous processing supply models,^6^,^7^ models that require production to operate at near optimal levels “by-design,” or whether this very requirement will in itself help drive the efficiency improvements demanded by patients and payers alike.
